# Addressing the Stroke Triage Challenge

**DOI:** 10.3389/fneur.2021.670204

**Published:** 2021-04-15

**Authors:** Rajiv Advani

**Affiliations:** ^1^Stroke Unit, Department of Neurology, Oslo University Hospital, Oslo, Norway; ^2^Neuroscience Research Group, Stavanger University Hospital, Stavanger, Norway

**Keywords:** stroke, paramedic (doctor's assistant), triage – emergency service, ambulance, thrombolytic, thrombectomy, education, acute stroke

The treatment of acute ischemic stroke (AIS) has undergone a revolution. More than two decades have passed since the use of intravenous thrombolysis (IVT) was approved for use within 4.5 h of symptom onset (1). The phrase “Save a minute, save a day” has been used to describe the profound effect of time savings where IVT is administered (2).

Newer works have shown that IVT can also be effective in selected patients up to 9 h after the onset of symptoms (3).

More severe ischemic strokes caused by a large vessel occlusion (LVO) are associated with significantly higher morbidity and mortality rates. Successful Mechanical Thrombectomy (MT) for LVO stroke within 6 h has a number needed to treat (NNT) for an improvement in clinical outcome as low as 2.6 (4). Based on newer trials in 2015 the time frame for MT in patients with a LVO stroke was expanded to 8 h from symptom onset (5). Later the same year, this time frame was expanded to 12 h (6). The latest in this succession of MT trials has shown that carefully selected patients with LVO can benefit from treatment up to 24 h after symptom onset (7). These randomized controlled trials selected patients using advanced radiological imaging to determine the presence of viable penumbra.

Despite the expansion of the treatment window early treatment is crucial. If the time saving in the setting of IVT wasn't profound enough, saving a minute prior to treatment in the setting of Mechanical Treatment (MT) grants a week of disability free life (8). This time saving has now been quantified and puts even more emphasis on efficacious treatment – “Save a minute, Save a week”. In setting of acute stroke treatment every minute counts. The afore mentioned trials have shown that a significant number of patients achieve functional independence (mRS 0–2) (6, 9) with the vast majority requiring only modest assistance (mRS 0–3) (10). Time to treatment has been shown to be the key factor associated with better clinical outcome in a large metanalysis (10). The clinical outcomes are significant for each individual patient, but also have a greater socioeconomic impact. The annual cost of care owing to residual stroke morbidity is as great as 90,000 USD, whereas patients achieving functional independence have a significantly lower cost of care, around 15,000 USD (11).

This paradigm shift in treatment has led to an increased burden of duty on paramedics and emergency medical services (EMS) worldwide. The focus being firmly placed on rapid triage and transport of these patients to appropriate stoke treatment centers. Traditionally the FAST (Face, Arms, Speech, Time) acronym has been used to detect a suspected stroke (12). FAST, as a pre-hospital triage tool, has low sensitivity for the detection of a LVO stroke. This has led to the development more specialized stroke triage scales (13, 14). The aim of these newer scales has been to more accurately detect LVO stroke and triage patients to centers offering MT, avoiding unnecessary delay at primary treatment centers. Triaging patients with more severe strokes to comprehensive treatment centers where MT can be performed has been shown to be effective (15). A plethora of these pre-hospital triage scales have been developed showing similar accuracy (16). Centers where Emergency Medical Services (EMS) services employ the use of these newer triages scales have shown time savings and clinical benefit for those patients requiring MT (17).

These newer pre-hospital scales have however only been put into clinical practice at a limited number of pre-hospital services. In addition to a lack utilization of newer pre-hospital triage scales the time window for urgent triage must be addressed. The majority of triage systems used by the EMS in Scandinavia and Europe prioritize acute ischemic stroke symptoms as urgent within 6 h of symptom onset (18, 19). This, based on the AHA/ASA recommendations, is also the case for severity-based triage used across the USA (20).

This shortened time frame excludes patients eligible for rapid reperfusion therapy from 6 to 24 h of symptom onset; precluding more than 50% of the therapeutic window (18). Similarly, patients with unknown symptom onset or wake up strokes are also not always triaged as urgent. These patients potentially stand to benefit greatly from reperfusion therapy (21).

Mobile Stroke Units (MSU) are an innovative strategy employed with great success in some countries with improved stroke treatment times and clinical outcomes (22). However, this strategy relies on large ambulances with trained personnel being able to effectively access the patient population (23). Furthermore, the cost-effectiveness of this initiative is yet to be established for more generalized use (24). Ultimately, with or without access to MSU, symptom recognition, and correct EMS triage are paramount.

EMS worldwide should aim to implement advanced triage scales into clinical practice. In recent years paramedics have successfully used more advanced diagnostic scales. Some examples of these scales, amongst many others, include CPSSS (25), RACE (14), and ACT-FAST (26). Their use has been validated in the setting of LVO diagnostics; showing an excellent degree of agreement between doctors and paramedics (26). This will not only augment the detection of a suspected stroke, but also help guide the patient to the appropriate treatment center and treatment pathway. The new expanded treatment windows for MT in the setting of LVO stroke should also be implemented into clinical practice. Limiting the urgent triage response to 6 h significantly limits the therapeutic window for these patients and eventual reperfusion therapy. These two factors in combination warrant an overhauling of current pre-hospital stroke triage guidelines.

These newer triage routines have the potential to be implemented in emergency departments in small hospitals where comprehensive stroke treatment isn't offered. Patients showing symptoms of LVO stroke based on the use of newer stroke triage scales arriving at hospitals where MT isn't offered could be transferred directly to comprehensive stroke centers. This would mean that a patient needing MT wouldn't need to undergo a primary evaluation at one hospital before being transferred to a nearest comprehensive stroke center, reducing significant delays before recanalization.

Newer triage routines are being implemented too slowly and patients worldwide are being precluded from treatment daily. The mainstay of this implementation should be based on paramedic education and updating of existing triage algorithms. A better understanding of the recent advances in stroke treatment and implementation of newer pre-hospital triage scales could offer potentially life-saving treatment to many more patients.

## Author Contributions

The author confirms being the sole contributor of this work and has approved it for publication.

## Conflict of Interest

The author declares that the research was conducted in the absence of any commercial or financial relationships that could be construed as a potential conflict of interest.
